# Innovative modified light bulb technique with custom offset curettes for treating precollapsed femoral head osteonecrosis

**DOI:** 10.1093/jhps/hnaf021

**Published:** 2025-06-12

**Authors:** Ali Parsa, Morteza Behjat, Neda Mirzaei, Mohammad Ghorbani, Elham Rahmanipour

**Affiliations:** Orthopedic Research Center, Department of Orthopedic Surgery, Mashhad University of Medical Sciences, Mashhad, Iran; American Hip Institute, Chicago, Illinois, United States; Department of Orthopaedics Surgery, School of Medicine, Rasoul Akram Hospital, Iran University of Medical Sciences, Tehran, Iran; Orthopedic Research Center, Department of Orthopedic Surgery, Mashhad University of Medical Sciences, Mashhad, Iran; Orthopedic Research Center, Mashhad University of Medical Sciences, Mashhad, Iran; Immunology Research Center, Mashhad University of Medical Sciences, Mashhad, Iran

## Abstract

Osteonecrosis of the femoral head is a devastating disease that accounts for 10–15% of all hip arthroplasty cases in the USA, eventually leading to femoral head collapse and joint destruction. Even the current forms of treatment only slightly slow down the progression of the disease itself. This article describes a modified lightbulb technique, which perfects the method, the instrument, and the flap used in order to ensure hip musculature preservation and improved outcomes. With the direct anterior approach, there is no need to perform surgical dislocation, so the time of the surgery is reduced, as well as the risk of vascular injury. The necrotic bone can be precisely removed using custom-made offset curettes, and a bone graft substitute and autograft can be placed under C-arm guidance. This technique may be beneficial in treating early osteonecrosis of the femoral head by reducing operative time and reducing the risk of complications related to surgical dislocation.

## Introduction

Osteonecrosis of the femoral head is a progressive disease [1]. Once symptoms appear, the condition typically advances towards femoral head collapse and eventual hip joint failure [2]. This disease accounts for 10–15% of all hip arthroplasty cases in the USA [1], primarily affecting individuals in their third to fifth decades of life who are often active members of society [1].

Currently, no approved treatment method has fully succeeded in halting the progress of the disease before femoral head collapse [2]. The lightbulb technique, first described by Seyler *et al*. [3], introduced a novel approach for treating early-stage osteonecrosis by enabling complete removal of necrotic bone and precise placement of a bone graft [3].

This article aims to present the modified lightbulb technique with alterations in the approach, device, and graft used to preserve the muscles in the hip area and achieve improved treatment outcomes. In the initial technique described by Seyler *et al*., the approach to the hip joint is from the anterolateral side [3]. In the direct anterior approach (DAA), the incision and surgical pathway are entirely muscle-sparing, with no muscle removal [4]. In contrast, the anterolateral approach, which is closer to the abductor muscles, may result in some degree of the Gluteus medius muscle fibre damage due to traction [3, 4]. By contrast, the DAA is entirely muscle-sparing, as it avoids any muscle removal during the procedure. Our modified method aims to achieve the same level of access while preserving all musculature by utilizing a DAA [4].

Given that the access point for the necrotic lesion is at the femoral head-neck junction—and considering the need to navigate femoral head anteversion to reach the typical necrosis site, which is often anterolateral and anterior—we designed specialized offset curettes for this technique [5, 6]. These instruments, inspired by J-shaped ENT curettes [6], feature a longer handle and a slightly modified curvature to enhance precision during debridement.

## Surgical technique

**Figure 1.**
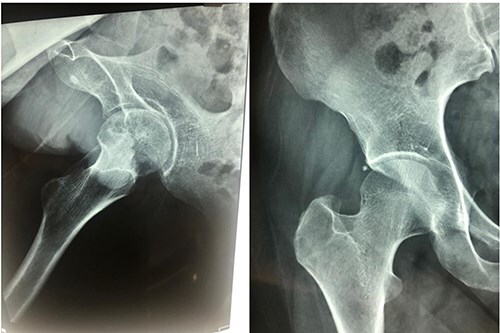
The preoperative radiographs showing moderate degree of osteonecrosis of femoral head.

**Figure 2.**
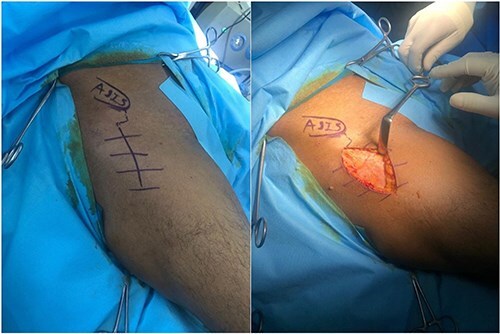
The skin incision of direct anterior approach made from lateral to anterior superior iliac spine towards distal and lateral in alignment of tensor fascia lata muscle.

The lightbulb method is performed with the patient in the supine position on a standard operating room table. A preoperative radiograph is obtained (Fig. 1). The incision is made 1 cm distal and 2 cm lateral to the anterior superior iliac spine, extending approximately 6 cm in a slightly oblique direction, aligned with the centre of the tensor fascia lata muscle (Fig. 2).

**Figure 3.**
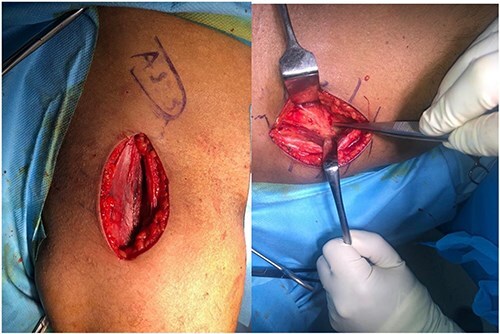
The direct anterior approach is via tensor fascia lata and rectus femoris and between them the joint capsule is revealed.

**Figure 4.**
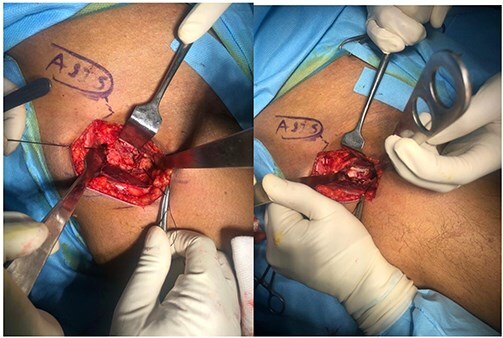
The incision of joint capsule and exposure of anterior part of femur neck.

After incising the skin and subcutaneous tissue, the fascia is opened, and the tensor fascia lata muscle is retracted laterally. Using the DAA [7], the hip joint capsule is exposed by accessing the interval between the rectus femoris (medially) and the tensor fascia lata (laterally) (Fig. 3). A potential risk of this approach is injury to the lateral femoral cutaneous nerve [8]. Once the capsule is reached, pericapsular fat is carefully removed using a rongeur to improve visualization of the capsule’s boundaries. An L-shaped capsulotomy is then performed parallel to the lateral edge of the femoral neck, exposing the femoral neck and the base of the femoral head (Fig. 4).

Next, a 1 × 2 cm cortical bone window is created in the anterior femoral neck using a micro-oscillating saw and osteotome, guided by preoperative radiographs and MRI to target the area of maximal necrosis. The extracted bone plug is preserved on a saline-soaked gauze for later use.

**Figure 5.**
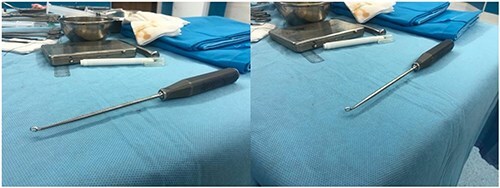
Custom offset curettes specialized for lightbulb shape of femur head.

A fine, mushroom-tipped burr is used to create a channel into the femoral head (Fig. 5). Under C-arm guidance, a custom offset curette is manoeuvered to the necrotic area identified in preoperative imaging, ensuring precise debridement while avoiding cartilage penetration. The resulting cavity, which is in a lightbulb shape, is thoroughly cleared of necrotic bone, and samples may be sent for pathological confirmation.

**Figure 6.**
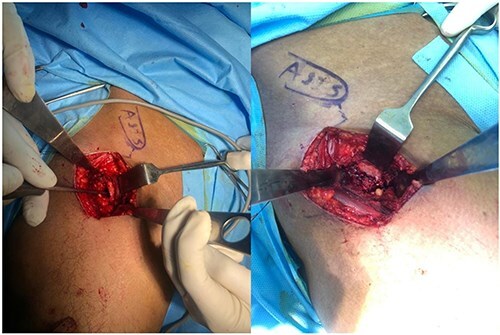
The curettage of femoral head necrosis and placing allograft and autograft.

Following curettage, the cavity is typically larger than the initial necrotic area. It is then irrigated and filled with a mixture of autograft and bone graft substitute (Fig. 6). The capsule is meticulously repaired, and after local anesthetic infiltration, the fascia, subcutaneous tissue, and skin are closed without the use of a drain.

**Figure 7.**
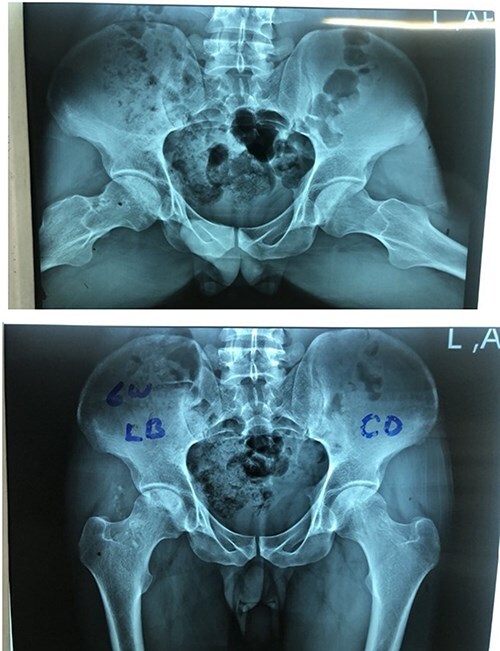
The 6 week postoperative radiographs comparing lightbulb procedure in the right side and core decompression in the left side.

A postoperative radiograph at the 6-week follow-up comparing the modified lightbulb technique to core decompression is shown in Fig. 7.

## Discussion

The lightbulb procedure, which involves debridement of necrotic bone and bone grafting of the femoral head, traditionally requires surgical hip dislocation to access the affected area [3]. This approach allows for direct visualization, which is crucial for accurate debridement and graft placement [5].

Seyler *et al*. [3] were the first to evaluate this technique, studying 33 patients (39 hips) with Ficat-Arlet stage II or III osteonecrosis treated using the lightbulb method with BMP-7 supplementation. At an average follow-up of 36 months, the mean Harris Hip Score (HHS) improved from 50 to 75 points, with 67% of hips avoiding reoperation.

Rosenwasser *et al*. [9] followed 13 patients (15 hips) with avascular necrosis of the femoral head for a mean of 12 years after lightbulb surgery. The majority (87%) remained asymptomatic with minimal osteoarthritis progression, while 13% required revision surgery. No cases of infection, femoral neck fractures, or thromboembolic events were reported, though heterotopic ossification occurred in two patients.

Mont *et al*. [10] evaluated 19 patients (21 hips) with a minimum follow-up of 48 months post-lightbulb surgery. Clinical success, based on HHS outcomes, was achieved in 86% of cases. Similarly, Marker *et al*. [5] analysed 126 hips, comparing 41 treated with the lightbulb technique (augmented with lyophilized type I collagen or recombinant human BMP-7) to 79 hips treated with multiple drilling. Both groups demonstrated significant pain reduction following surgery.

Hip dislocation for surgical access is typically performed via the anterolateral approach, with technique variations depending on the surgeon’s preference and patient anatomy [3]. More recently, the DAA has gained popularity in hip replacement surgery due to its muscle-sparing benefits. In light of this, we sought to refine the lightbulb procedure by adopting a more muscle-sparing approach. This modification allows for access to the femoral head-neck junction without the need for surgical dislocation, facilitating complete debridement of osteonecrosis lesions within the Osteonecrosis of femoral head region.

The primary advantage of this modified approach is the elimination of surgical dislocation. By using custom-made curettes specifically designed for this procedure, full debridement of the necrotic area can be achieved without direct visualization, relying solely on C-arm guidance. This results in a significantly less invasive procedure, reducing both operative time and the risk of vascular damage to the femoral head and neck. While long-term clinical data is still needed, this technique is expected to lower the risk of vascular injury. However, this technique has limitations. Creating a cortical window in the femoral neck may increase mechanical stress, potentially raising the risk of fracture. Though rare and unobserved in our patients, this remains a theoretical concern, requiring careful assessment of bone quality and patient risk factors.

Traditionally, the lightbulb procedure has been performed using the anterolateral approach. However, this study is the first to introduce a modified version utilizing the DAA, offering potential benefits such as reduced surgical time and faster postoperative recovery. To date, all prior modifications of the lightbulb technique have adhered to the anterolateral approach.

Additionally, this study introduces the use of J-shaped curettes, originally designed for ENT surgeries, to address the curvature of the femoral head and neck during debridement. These specialized curettes allow for the removal of larger necrotic areas compared to standard curettes, representing another key innovation in this procedure.

In the standard Mont method, iliac crest bone autograft is used to fill the femoral head cavity. In contrast, our modified lightbulb technique employs a combination of iliac crest autograft and bone substitute allograft to enhance graft integration and structural support [10].

Furthermore, we conducted a retrospective cohort analysis to compare the clinical outcomes of femoral head decompression using the modified lightbulb technique versus the multiple drilling method. Clinical evaluations, based on Visual Analogue Scale and Hip Disability and Osteoarthritis Outcome Score criteria, demonstrated that the modified lightbulb method was significantly superior to multiple drilling in both immediate postoperative outcomes and follow-ups at 6 and 12 months postsurgery [11].

To date, core decompression remains the most widely used treatment for femoral head osteonecrosis, largely because it is straightforward and can be performed by most surgeons [12]. More advanced techniques, such as the light bulb procedure, have historically been considered complex and time-consuming, limiting their widespread adoption [12]. However, we believe that this modified technique, being significantly less invasive and more efficient, has the potential to gain broader acceptance among surgeons.

## Data Availability

No new data were generated or analysed in support of this research.
